# Sourcing Vanillin *via* Fermentative Biotechnology

**DOI:** 10.17113/ftb.64.01.26.9445

**Published:** 2026-02-15

**Authors:** Stefanie Schmid, Beate Berchtold, Harald Pichler

**Affiliations:** 1 Institute of Molecular Biotechnology, Graz University of Technology, Petersgasse 14, 8010 Graz, Austria; 2 Austrian Centre of Industrial Biotechnology (acib GmbH), Krenngasse 37, 8010 Graz, Austria; 3 NAWI Graz, Mozartgasse 12/2, 8010 Graz, Austria; 4 BioTechMed Graz, Mozartgasse 12/2, 8010 Graz, Austria

**Keywords:** vanillin, biotechnology, recombinant hosts, biotransformation, vanillin toxicity, *in situ* product removal

## Abstract

Less than 1 % of the annual worldwide consumption of vanillin can be met by extracting the aromatic compound from vanilla (*Vanilla planifolia*) pods. For 150 years, vanillin has also been derived through chemical synthesis, which remains the main source (>80 %) of vanillin today, despite growing environmental concerns due to considerable chemical waste disposal issues. ‘Natural’ vanillin is in high demand for flavour and fragrance applications. Thus, biotechnological routes using an array of recombinant hosts have been devised to obtain vanillin through fermentation of natural precursors, *e.g*. ferulic acid, (iso)eugenol and glucose. These processes, often classical biotransformations, result in ‘natural’ vanillin according to European and US legislation. A significant technical hurdle in fully fermentative routes is vanillin toxicity, which impairs cellular proliferation at relatively low, *i.e*. commercially uninteresting, vanillin concentrations. In addition to adopting the plant-derived solution, *i.e*. product glycosylation, to sequester and store vanillin glycosides, sophisticated *in situ* product removal strategies have been used to obtain industrially relevant amounts of ‘natural’ vanillin.

## INTRODUCTION - VANILLIN

Vanillin, an aromatic aldehyde (4-hydroxy-3-methoxybenzaldehyde, C_8_H_8_O_3_), is the primary aroma compound of the vanilla orchid (*Vanilla planifolia*), making up mass fraction of 1–2 % in vanilla pods (*1*). Commercial vanillin is a crystalline white to slightly yellow powder with a sweet vanilla smell. It has a wide variety of industrial applications, spanning from perfumes (as a fragrance) and food (as a flavour component) to pharmaceuticals (as an intermediate compound or odour-masking agent). Global vanillin production reached approx. 60 000 tonnes in 2024 (*2*, *3*).

### Classification of vanillin according to origin

#### Natural vanillin

In 1858, vanillin was first isolated from vanilla pod extracts by Theodore Nicolas Gobley, who then identified its chemical structure (*4*). Vanilla beans originated in Mexico; however, according to the Food and Agriculture Organisation's global statistical database (*5*), they are now most commonly produced in Madagascar, Indonesia, Mexico and China. The vanilla orchid is cultivated in moist, warm, tropical climates. Fresh vanilla beans have a rather unpleasant scent and develop the typical sweet smell only upon curing. The beans contain around 20 g of vanillin per kg dry mass, stored as vanillin glycosides. The characteristic, rich ʹvanillaʹ smell is composed of more than 200 molecules; however, vanillin contributes mainly to the characteristic scent (*2*, *6*, *7*). Currently, less than 1 % of the globally produced vanillin is harvested from the vanilla plant itself, as the process is costly and dependent on the plant's natural development cycle (*3*). To produce 1 kg of purified vanillin, roughly 500 kg of vanilla pods are required, necessitating the pollination of around 40 000 vanilla orchid flowers (*5*). Moreover, vanillin is prone to oxidation and degradation due to reactions with other compounds, which lower the overall quality and purity by forming unwanted side products. This necessitates effective purification steps to obtain pure natural vanillin (*8*). Therefore, vanilla beans are a low-yield source of vanillin, and it is impossible to meet the market demand in this way. Around 80 % of global vanillin is produced *via* chemical synthesis, and the rest *via* biosynthetic pathways. The price of chemically synthesised vanillin is only 1 % that of natural vanillin (*2*, *3*, *9*).

#### Chemical vanillin

In 1875, less than 20 years after its first isolation from the vanilla bean, synthetic vanillin became commercially available in France and the United States, sold for $176 per kg (*10*). It was obtained by the isomerisation of eugenol, followed by an oxidation step (*11*). Nowadays, chemical vanillin is produced from aromatic compounds, such as eugenol, guaiacol (*10*) and lignin (*4*). While synthetic vanillin is similar to its natural counterpart, it does vary in flavour and smell. This is due to the absence of interactions with associated compounds found in the vanilla bean, through which the full vanilla flavour profile emerges (*12*). Additionally, chemical vanillin is restricted in some industrial sectors due to concerns about health risks from racemic mixtures (*13*). The production of chemical vanillin not only involves hazardous chemicals, but it also generates substantial waste, with lignin-derived processes requiring the safe removal of 160 kg of waste per kg of vanillin. This has resulted in the closure of lignin-derived vanillin production in some regions due to environmental concerns (*4*, *10*).

#### Bio-vanillin

Microbial-based production methods offer a promising approach to address the increasing demand for sustainable and economically viable vanillin production. Vanillin produced from natural precursors such as lignin, ferulic acid, isoeugenol, eugenol and glucose through microbial fermentation is categorised as ’natural’ by the European and US food legislation (*4*, *12*, *14*, *15*). Researchers have identified several microbial methods for vanillin production, each with advantages and limitations. To ensure the process is economically feasible, it is essential to identify cost-effective and readily available precursors. Among these, *de novo* biosynthesis from simple carbon sources, particularly glucose, has gained significant interest. With a price of less than $0.30 per kg, glucose is a highly economical and readily available substrate, making it a favourable choice for vanillin biosynthesis. Furthermore, glucose is preferred as a substrate to ferulic acid, eugenol and other phenolic compounds as it is non-toxic to microorganisms (*16*–*18*). Lignin is another substrate of interest, as it is one of the most abundant polymers on earth and is produced in large quantities (millions of tonnes) as waste in the pulp and paper industry (*19*, *20*). For use as a food additive, the recombinant vanillin host organism must be generally recognised as safe (GRAS) by the US Food and Drug Administration (FDA) and/or have qualified presumption of safety (QPS) status according to the European Food Safety Authority (EFSA) (*21*).

## BIOTECHNOLOGICAL PRODUCTION OF VANILLIN

### Bacterial vanillin synthesis

**Fig. 1:**
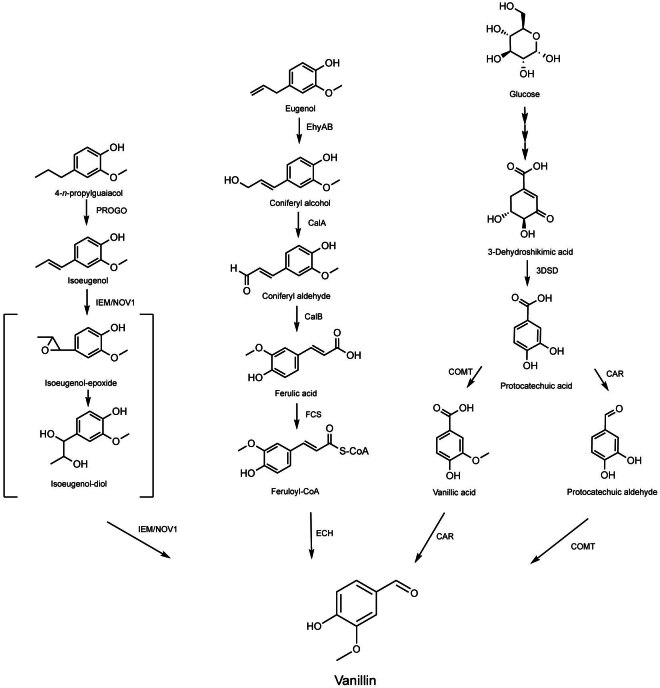
**‘**Natural’ vanillin production routes from the precursors ferulic acid, (iso)eugenol and glucose. IEM=isoeugenol monooxygenase, EhyAB=eugenol hydroxylase, CalA=coniferyl alcohol dehydrogenase, CalB=coniferyl aldehyde dehydrogenase, FCS=feruloyl-CoA synthetase, ECH=enoyl-CoA hydratase/aldolase, 3DSD=3-dehydroshikimate dehydratase, COMT=catechol-O-methyltransferase, CAR=carboxylic acid reductase, PROGO=4-propylguaiacol oxidase, NOV1=isoeugenol dioxygenase

As a model organism, *Escherichia coli* has been genetically engineered to produce a vast number of compounds (*22*–*24*), including vanillin (*9*). In 2005, Yoon *et al*. (*25*) introduced a feruloyl-CoA synthetase (*fcs*) and enoyl-CoA hydratase/aldolase (*ech*) from *Amycolatopsis* sp. into *E. coli via* a plasmid (Fig. 1 and Table 1 (*25*-*39*)). Using ferulic acid as a carbon source, the engineered strain produced 1.1 g/L vanillin (*25*). Enhancing the expression of the citrate synthase gene (*gltA*) of the tricarboxylic acid (TCA) cycle resulted in 1.98 g/L vanillin from 3 g/L ferulic acid (*26*). To further increase the yield, the consumption of acetyl-CoA was optimised by activating the glyoxylate bypass through the deletion of *icdA* gene. Amplification of the *gltA* gene and deletion of the *icdA* gene, together with the use of a polystyrenic resin XAD-2 to reduce vanillin toxicity, yielded 5.14 g/L vanillin with a molar conversion rate of 86.6 % (*26*).

**Table 1:** Recombinant bacterial hosts and their vanillin yields

Strain	Modification	Substrate	*Y*(vanillin)/(g/L)	Reference
*E. coli* DH5α	Integration of FCS and ECH	Ferulic acid	1.1	(*25*)
*E. coli* DH5α	Integration of FCS and ECH, amplification of *gltA*	Ferulic acid	1.98	(*26*)
*E. coli* DH5α	Integration of FCS and ECH, amplification of *gltA, ΔicdA*	Ferulic acid	5.14	(*26*)
*E. coli* K12 MG1655	Integration of CAR and COMT, deletion of AKRs and ADHs	Glucose	0.481	(*27*)
*E. coli* BL21(DE3)	Integration of IEM (from *P. putida* IE27)	Isoeugenol	28.3	(*28*)
*E. coli* BL21(DE3)	Integration of IEM (from *P. nitroreducens *Jin1)	Isoeugenol	38.34	(*29*)
*E. coli* NEB 10β and *E. coli* BL21 AI	Integration of PROGO and NOV1	4-*n*-propylguaiacol (from raw spruce)	66 % [sic!]	(*30*)
*E. coli* MG1655 RARE	Integration of TPADO, DCDDH, CAR, COMT	Terephthalic acid	0.119	(*31*)
*P. putida* KT2440	Integration of FCS and ECH, *Δvdh*	Ferulic acid	1.31	(*32*)
*Amycolatopsis* sp. ATCC 39116	Integration of FCS and ECH	Ferulic acid	22.3	(*33*)
*P. fluorescens* BF13	Integration of FCS and ECH, *Δvdh*	Ferulic acid	1.28	(*34*)
*P. acidilactici* BD16	Integration of FCS and ECH	Ferulic acid	0.48	(*35*)
*P. acidilactici* BD16	Integration of FCS and ECH	Ferulic acid (from agro-biomass waste)	4.01	(*36*)
*Streptomyces *sp*.* V-1	Strain isolated from soil, use of resin DM11	Ferulic acid	19.2	(*37*)
*C. glutamicum*	Integration of CAR and COMT, Δ*pcaHG*, Δ*vanAB*, and Δ*NCgl0324*	Glucose	0.31	(*38*)
*P. resinovorans* SPR1	Screening for eugenol degrading bacteria	Eugenol	0.24	(*39*)

FCS=feruloyl-CoA synthetase, ECH=enoyl-CoA hydratase/aldolase, CAR=carboxylic acid reductase, COMT=catechol-O-methyltransferase, IEM=isoeugenol monooxygenase, AKR=aldo-keto reductase, ADH=alcohol dehydrogenase, PROGO=4-propylguaiacol oxidase, NOV1=isoeugenol dioxygenase, TPADO=terephthalate 1,2-dioxygenase, DCDDH=2-dihydroxy-3,5-cyclohexadiene-1,4-dicarboxylate dehydrogenase, vdh=vanillin dehydrogenase, *pcaHG*=protocatechuate dioxygenase, *vanAB*=vanillate demethylase subunits A and B, *NCgl0324*=aromatic aldehyde reductase from the bacterium *Corynebacterium glutamicum*

Due to its low price and availability, glucose is considered a more cost-effective carbon source than ferulic acid (*40*). In 1998, Li and Frost (*41*) developed a microbial biosynthetic pathway for vanillin production from glucose. In this pathway, *E. coli* was engineered to catalyse the dehydration of 3-dehydroshikimic acid and the regioselective methylation of the resulting protocatechuic acid to produce vanillic acid. Vanillic acid was then reduced to vanillin by aryl-aldehyde dehydrogenase, purified from *Neurospora crassa* lysate. While effective, the *in vitro* reduction step depended on expensive cofactors (adenosine triphosphate (ATP) and nicotinamide adenine dinucleotide phosphate (NADPH)), making the process economically unfeasible for large-scale production. In 2024, Wu *et al*. (*27*) optimised the protocatechuic acid pathway in *E. coli* K12 MG1655 by screening for highly active carboxylic acid reductases and catechol O-methyltransferases. Using this vanillin production route can lead to the formation of vanillyl alcohol as a major side product. Therefore, the authors deleted three endogenous aldo–keto reductases and three alcohol dehydrogenases, while also regulating competitive metabolic pathways. Vanillin was produced from glucose at a yield of 481.2 mg/L (*27*).

Using isoeugenol monooxygenase (IEM), vanillin can be produced in one step from isoeugenol, a compound found in several essential oils, *e.g.* derived from clove, nutmeg or cinnamon (*42*). A recombinant plasmid carrying the IEM gene from *Pseudomonas putida* IE27 under the control of the T7 promoter was introduced into *E. coli* BL21(DE3), yielding 28.3 g/L vanillin from 230 mM isoeugenol. This represents a molar conversion yield of 81 % after 6 h of reaction and no accumulation of side products such as vanillic acid or acetaldehyde was reported (*28*). In 2021, the IEM from *Pseudomonas nitroreducens* Jin1 was cloned into *E. coli,* resulting in a recombinant strain that produced 38.34 g/L vanillin with >99 % purity (*29*). Marić *et al.* (*30*) presented a strategy to convert the lignin-derived monomer 4-*n*-propylguaiacol (4PG) into vanillin using engineered *E. coli* strains expressing 4-propylguaiacol oxidase (PROGO) and isoeugenol dioxygenase (NOV1). To overcome substrate inhibition, *i.e.* 4PG competitively inhibiting the second-step enzyme, and to accommodate different temperature optima (37 °C for PROGO and 25 °C for NOV1), a stepwise one-pot strategy was established. Using this method, a vanillin yield of 66 % from raw spruce-derived lignin oil was achieved on a small laboratory scale (250 µL reactions).

Concomitantly, to address plastic waste pollution, a novel approach to vanillin production has been explored. An *E. coli* MG1655 RARE strain was engineered to carry a novel synthetic pathway that can produce vanillin from polyethylene terephthalate (PET). Achieving a 79 % conversion of terephthalic acid to vanillin (119 mg/L), the study proposed a 157-fold improvement in vanillin yield through process optimisation, *i.e.* reaction temperature, medium optimisation and *in situ* product removal. This work documents the first biological upcycling of post-consumer PET into a single value-added small molecule. The findings suggest that PET waste may serve as a carbon source for producing high-value chemicals, contributing to a more circular economy and reducing plastic pollution (*31*).

Beyond *E. coli,* various other bacteria have also been used for vanillin production (Table 1). *Pseudomonas putida* KT2440 was optimised to convert ferulic acid to vanillin by enhanced chromosomal expression of the genes *fcs* and *ech*. To decrease further vanillin metabolism, Graf and Altenbuchner (*32*) deleted the vanillin dehydrogenase gene (*vdh*); however, this did not fully prevent vanillin turnover. High initial conversion rates and molar vanillin yields of up to 86 % were achieved within just 3 h, with little by-product formation. The highest vanillin titre reached in this approach was 1.31 g/L (*32*). *Amycolatopsis* sp. ATCC 39116 is attractive as a vanillin production host due to its high vanillin tolerance. After the introduction of *fcs* and *ech* through a plasmid, deletion of *vdh* and an optimisation of ferulic acid feeding strategies, a vanillin yield of 22.3 g/L was achieved. The deletion of *vdh* led to a more than 90 % decrease in vanillin turnover (*33*). A similar engineering strategy was used for converting ferulic acid to vanillin with *Pseudomonas fluorescens*, yielding 1.28 g/L (*34*).

Lactic acid bacterium *Pediococcus acidilactici* BD16 produced 0.48 g/L vanillin from 0.16 g/L [sic!] ferulic acid per milligram of recombinant cell biomass within 20 min of biotransformation. Heterologous genes *fcs* and *ech* were introduced *via* plasmids, and the process was statistically optimised and scaled up (*35*). The same recombinant *P. acidilactici* strain was used to explore vanillin production from ferulic acid in rice bran, targeting a more economical vanillin production. The strain yielded 4.01 g/L vanillin within 24 h of incubation with rice bran medium (*36*).

To address the problem of vanillin toxicity and product inhibition, the ability of resins to adsorb vanillin *in situ* was tested during the bioconversion of ferulic acid to vanillin using *Streptomyces* sp. strain V-1, which was isolated from soil samples and characterized. Using resin DM11, the highest amount of vanillin and the lowest amount of ferulic acid were adsorbed, resulting in a yield of 19.2 g/L vanillin within 55 h (*37*).

Several enzymes involved in reducing aromatic aldehydes to their corresponding alcohols were identified in *Corynebacterium glutamicum.* After the deletion of *pcaHG*, *vanAB* and *NCgl0324* and the introduction of a carboxylic acid reductase and mutated catechol O-methyltransferase, the strain produced 0.31 g/L vanillin from glucose (*38*). Using eugenol as a carbon source, Ashengroph *et al*. (*39*) screened for eugenol-degrading bacteria and found *Pseudomonas resinovorans*. Without further optimisation, resting cells of *P. resinovorans* SPR1 produced 0.24 g/L vanillin, *i.e.* a molar yield of 10 %.

### Fungal vanillin synthesis

In 1996, Lesage-Meessen *et al*. (*43*) described a two-step process using different filamentous fungi, *Aspergillus niger* and *Pycnoporus cinnabarinus*, to convert ferulic acid to vanillin (Table 2 (*17*, *18*, *43*, *44*, *47*-*51*)). First, *A. niger* transformed ferulic acid to vanillic acid, then *P. cinnabarinus* reduced vanillic acid to vanillin. Vanillic acid was produced at a molar yield of 88 %; however, the reduction to vanillin only yielded 22 %. Low vanillin yields resulted from *P. cinnabarinus* predominantly producing methoxyhydroquinone from vanillic acid. After the addition of phenolic precursors, the final vanillin yield was 237 mg/L (*43*). Following optimisation of medium components (*i.e.* carbon and nitrogen), environmental factors (such as pH), and a one-step biotransformation process under statistically optimal conditions, the molar yield of *P. cinnabarinus* increased to 54 %, producing 126 mg/L vanillin (*43*). Screening for isoeugenol-tolerant yeasts from soil samples led to the identification of *Trichosporon asahii.* This strain effectively converted isoeugenol to vanillin in a resting cell biotransformation without any genetic modifications. *T. asahii* produced 2.4 g/L vanillin from 5 g/L isoeugenol (*44*). Van den Heuvel *et al.* (*45*) demonstrated the potential of the flavoprotein vanillyl alcohol oxidase (VAO) from *Penicillium simplicissimum* for the ‘natural‘ synthesis of vanillin from creosol and capsaicin. The study identified two primary enzymatic pathways: (*i*) a two-step oxidative hydroxylation of creosol and (*ii*) the deamination of vanillyl amine (derived from capsaicin hydrolysis). While the capsaicin route was highly efficient, achieving nearly 100 % molar yields, the creosol route was limited by competitive inhibition and the formation of non-productive covalent flavin-substrate complexes at neutral pH.

**Table 2:** (Recombinant) fungal and plant hosts and their vanillin yields

Strain	Modification	Substrate	*Y*(vanillin)	Reference
*S. cerevisiae*	Integration of 3DSD, CAR, OMT	Glucose	0.045 mg/L	(*17*)
*S. pombe*	Integration of 3DSD, CAR, OMT	Glucose	0.065 mg/L	(*18*)
*P. cinnabarinus*	None	Ferulic acid	0.126 mg/L	(*43*)
*A. niger* I-1472 and *P. cinnabarinus* MUCL39532	None	Ferulic acid	0.237 mg/L	(*43*)
*Trichosporon asahii*	None	Isoeugenol	2.4 g/L	(*44*)
*S. cerevisiae* BY4741	Integration of 3DSD, CAR, OMT (in total 24 genetic modifications)	Glucose	0.366 mg/L	(*47*)
*Komagataella phaffii* GS115 *Δku70*	Integration of TAL, HpaB, HpaC, OMT, FCS, ECH	Glucose/caffeic acid	731.3 mg/L	(*48*)
*Capsicum frutescens*	Codon optimized *VpVAN*	Ferulic acid	0.057 %	(*49*)
*Ocimum sanctum*	*VpVAN* overexpression	Ferulic acid	1.98 mg/L	(*50*)
*Oryza sativa*	*Vp*VAN integration	Ferulic acid	544.72 μg/g	(*51*)

3DSD=3-dehydroshikimate dehydratase, CAR=carboxylic acid reductase, OMT=O-methyltransferase, TAL=tyrosine ammonia lyase, HpaB= 4-hydroxyphenylacetate 3-monooxygenase, HpaC=NAD(P)H-flavin oxidoreductase, FCS=feruloyl-CoA synthetase, ECH=enoyl-CoA hydratase/aldolase, VpVAN=vanillin synthase

To address the problems in the creosol pathway, a later study (*46*) used directed evolution to optimise VAO for vanillin production from creosol. While the wild-type enzyme was hindered by the formation of a stable, non-productive covalent flavin adenine dinucleotide (FAD) N-5-creosol adduct, a single round of error-prone polymerase chain reaction (PCR) followed by high-throughput screening identified seven mutants with enhanced activity. Among them, a variant with four amino acid exchanges (I238T, F454Y, E502G and T505S) showed up to a 40-fold increase in catalytic efficiency (*k*_cat_/*K*_m_) at pH 10. The improvement resulted from destabilization of an abortive adduct, which shifted the reaction towards product formation.

Hansen *et al*. (*17*) achieved the first successful microbial vanillin biosynthesis from glucose by integrating the complete vanillin biosynthesis pathway into a single microorganism. This was demonstrated in the yeasts *Saccharomyces cerevisiae* and *Schizosaccharomyces pombe*, with vanillin production reaching 45 and 65 mg/L, respectively. Key enzymes included 3-dehydroshikimate dehydratase from *Podospora pauciseta*, an aromatic carboxylic acid reductase from *Nocardia*, and an O-methyltransferase from *Homo sapiens*. These findings suggested that the engineered yeasts represent a sustainable alternative to petrochemically derived vanillin (*17*). More recently, researchers used a MARE (minimal aromatic aldehyde reduction) yeast platform to improve vanillin yield from glucose by minimising vanillin reduction to vanillyl alcohol, optimising cofactor supply and reconfiguring the yeast's central metabolism. Overall, a total of 24 genetic modifications were introduced into *S. cerevisiae*, yielding a final vanillin titre of 365.55 mg/L (*47*). Building on these advances in metabolic engineering, Guo *et al.* (*48*) reported the first successful *de novo* synthesis of vanillin in the yeast *Komagataella phaffii*, reaching a titre of 731.3 mg/L using glucose as the sole carbon source. To prevent vanillin conversion to vanillic acid and vanillyl alcohol by endogenous enzymes, the researchers performed a systematic combinatorial knockout of 14 oxidoreductase genes. Notably, deletion of *PAS14*, *PAS15* and *PAS21* led to an 11.1-fold increase in vanillin production. Further improvements were achieved by rewiring metabolic pathways to increase the intracellular supply of l-tyrosine, NADPH and S-adenosylmethionine (SAM). To enhance the activity of the rate-limiting enzyme in the pathway, caffeic acid O-methyltransferase (*Nt*COMT), Guo *et al.* (*48*) combined molecular docking with saturation mutagenesis to generate the *Nt*COMT^N312A/H315N^ variant. This variant exhibited a widened substrate channel and a 49.7 % increase in catalytic activity. Together, these strategies establish *K. phaffii* as a robust industrial chassis for the production of vanillin.

### Plant vanillin synthesis

Vanillin is produced in the pods of *V. planifolia* (*4*). In the plant, the enzyme vanillin synthase (*Vp*VAN) appears to convert ferulic acid to vanillin, which is glycosylated to protect the plant from the toxicity of the compound (*5*). In *Capsicum frutescens* (hot chilli pepper), ferulic acid and vanillin were found to be the intermediates in its phenylpropanoid biosynthetic pathway (*49*). Using biolistics, *C. frutescens* cells were transformed with a codon-optimised *VpVAN* (Table 2). For the biotransformation, immobilized cell cultures were used and the vanillin content of transformed calli was 0.057 % compared to 0.0003 % of untransformed calli (*49*). To influence the phenylpropanoid pathway and phenolic compound accumulation in *Ocimum sanctum*, the *VpVAN* gene was overexpressed *via Agrobacterium*-mediated transformation. LC–MS/MS analysis showed increased vanillin production in transgenic lines compared to *O. sanctum* wild type, with the highest vanillin content being (1.98±0.01) mg/g extract (*50*). A similar approach was used with rice calli derived from embryonic rice cells, engineered to carry a codon-optimised *VpVAN* gene. The vanillin yield in the fresh callus of rice cell culture was 544.72 μg/g (*51*). Compared to bacterial and fungal systems, plant vanillin synthesis shows lower yields and is also affected by growth environment and processing costs. At present, this makes recombinant plant hosts unsuitable to meet the vanillin market demand (*2*).

Microalgae are another versatile host for vanillin production, due to their rapid growth, high biomass productivity and metabolic versatility. They can produce several important molecules such as fatty acids, feedstocks for food, fuel and phenolic compounds (*52*, *53*). Rico *et al*. (*54*) showed that *Phaeodactylum tricornutum* growing in natural seawater under iron and copper stress could produce several precursors of vanillin biosynthesis pathways. *P. tricornutum* was able to produce protocatechuic, vanillic, caffeic, coumaric and ferulic acids (*54*). Cell cultures of *Haematococcus pluvialis* were studied for their biotransformation of ferulic acid, *p*-coumaric acid and coniferyl aldehyde. When immobilised, *H. pluvialis* produced 10.6 mg/L vanillin, 5.4 mg/L vanillic acid, 3.3 mg/L vanillyl alcohol, 1.6 mg/L protocatechuic acid, 1.1 mg/L *p*-coumaric acid and 1.4 mg/L *p*-hydroxybenzoic acid from these substrates (*55*). Following these results, Tazon *et al*. (*40*) hypothesised the presence of putative enzymes that could be used for vanillin biosynthesis. Based on the sequences of already characterised enzymes such as *Vp*VAN of *V. planifolia*, FCS and ECH of *Streptomyces* sp. V-1 and IEM of *Pseudomonas nitroreducens* Jin1, potential microalgal homologues were identified using BlastP in National center for biotechnology information (NCBI) (*56*). In four species of microalgae (*Chlamydomonas reinhardtii, P. tricornutum, H. pluvialis *and* Chlorella vulgaris*), homologues to the already characterised enzymes were identified. While microalgae are not yet ready for large-scale vanillin production, they represent an interesting alternative to the already existing hosts and may see increased use in the future.

## VANILLIN TOXICITY IN BIOTECHNOLOGICAL PRODUCTION

A major bottleneck in the biotechnological production of vanillin is product toxicity. Vanillin is known to have a toxic effect on many organisms, inhibiting growth at concentrations below 0.5 g/L in *S. cerevisiae* or ≥0.76 g/L in *E. coli* (*17*, *57*). As a rescue mechanism, the microorganisms rapidly oxidise or reduce vanillin to vanillic acid or vanillyl alcohol, respectively (*58*).

### Mechanisms of vanillin toxicity in microorganisms

In general, vanillin is considered a membrane-active compound capable of forming pores in membranes and thus, destroying the membrane integrity in *E. coli* (*59*, *60*). However, the underlying molecular mechanism of vanillin toxicity and its targets in the membrane remain unclear. A recent study suggested that adding exogenous vanillin disturbed key metabolic pathways in *E. coli,* leading to the accumulation of intracellular reactive oxygen species (ROS) and activation of stress response pathways (*58*). In *S. cerevisiae*, vanillin: (*i*) induced oxidative stress responses, possibly causing mitochondrial fragmentation (*61*), and (*ii*) repressed translation (*62*). However, the effects of endogenously produced vanillin may be different from those of externally added vanillin.

### Biotechnological strategies to mitigate vanillin toxicity

#### Modification via glycosylation

A more promising approach is the glycosylation of vanillin. By introducing a uridine diphosphate (UDP)-glycosyltransferase, vanillin was converted into vanillin-β-d-glucoside, which was non-toxic to yeast even at 25 g/L. In this study, a UDP-glycosyltransferase (UGT72E2) was expressed in *S. pombe* that was able to glycosylate 80 % of the produced vanillin (*17*). By further *in silico* design of *S. cerevisiae*, mutants with deletions of *PDC1* and *GDH1* produced up to 2-fold more vanillin glucoside than the unmutated strain in batch mode (*63*).

#### In situ product removal

Another strategy to mitigate vanillin toxicity is *in situ* product removal (ISPR), in which vanillin is continuously extracted from the fermentation broth using adsorbent resins or polymers. The product is then recovered, typically using organic solvents or distillation. For example, Ma and Daugulis (*64*) achieved a vanillin volumetric productivity of 0.45 g/(L·h) with *Amycolatopsis* sp. ATCC 39116 using Hytrel G4078W beads and ferulic acid as substrate, compared to 0.27 g/(L·h) in a single aqueous phase. However, the resin or polymer adsorbs vanillin, leading to losses during its recovery and requiring organic solvents. As an alternative, the same strain (*Amycolatopsis* sp. ATCC 39116) was cultivated in bioreactors using a multiple-pulse-feeding strategy with ferulic acid, where the broth was removed before each pulse and the biomass reused. This achieved a similar vanillin volumetric productivity of 0.46 g/(L·h) without a separate extraction phase (*65*). These studies highlight promising approaches for achieving high vanillin titres in non-engineered bacteria.

#### Metabolic engineering for tolerance and reduced degradation

Microbial strains can be engineered to increase their vanillin tolerance through chemical mutagenesis and adaptive evolution. An *S. cerevisiae* strain, EMV-8, grew in 2 g/L vanillin with a specific growth rate of *μ*=0.104/h, while its parent strain could not grow under the same conditions (*66*). Comparative genomic analysis between the vanillin-tolerant strain EMV-8 and its parent strain revealed more than 450 single nucleotide polymorphisms and 44 genes with insertions/deletions. Among the identified mutations, deleting the transcription factor YRR1 improved the maximum specific growth rate by 142 % in the presence of 0.91 g/L vanillin (*67*). Similarly, an nitrosoguanidine-mutagenesis-generated mutant of *E. coli* DH5α grew well in the presence of 2.0 g/L vanillin, while its wild-type strain showed only limited growth (*68*).

Microorganisms possess natural detoxification mechanisms involving the reduction of vanillin to vanillyl alcohol or its oxidation to vanillic acid. However, these are undesired side reactions in vanillin production as they lower the final vanillin yield. To decrease vanillin degradation, genes associated with these pathways are knocked out to increase vanillin titres. In *S. cerevisiae*, the alcohol dehydrogenase *ADH*6 (*YMR318C*) has been identified as one of the crucial genes for vanillin reduction (*17*, *69*). Deletion of *ADH6* decreased the conversion rate of vanillin to vanillyl alcohol by 50 % (*17*). Besides *ADH6*, several other gene products are associated with vanillin reduction activity, such as *YNL134C* or *YJR096W* (*70*). Recently, Mo and Yuan (*47*) developed a MARE platform strain for *S. cerevisiae* with 24 modifications by deleting a set of alcohol dehydrogenases, aldo-keto reductases and aldehyde reductases. This strain produced a vanillin titre of 365 mg/L from glucose with no detectable amount of vanillyl alcohol, highlighting the importance of eliminating competing degradation pathways.

## CONCLUSIONS

‘Natural’ vanillin can be produced from the precursors ferulic acid, (iso)eugenol and glucose employing diverse natural and engineered host strains. The volumetric vanillin yields from biotransformations based on aromatic precursors are apparently higher than those from fermentations with glucose as the substrate. Both approaches, biotransformation of aromatic precursors and *de novo* bioproduction of vanillin from glucose or related carbon sources, have their advantages and disadvantages. Thus, at present, there is no clear preference for one or the other route in sourcing ‘natural’ vanillin. Biotransformations require costly precursors but deliver higher volumetric yields. Glucose as a feedstock is cheaper than aromatic precursors, but the fermentation yields are limited by vanillin toxicity and the capacities of vanillin sequestration strategies. Developing sophisticated vanillin withdrawal strategies might shift the balance in favour of the fermentative approach in future work, particularly as scaling a glucose-based process should be commercially more straightforward than scaling biotransformations of aromatic precursors.
